# Supplementary material for: What does it take to learn the rules of RNA base pairing? A lot less than you may think
Source: bioRxiv. 2025 Aug 2:2025.07.31.668042. Preprint. [Version 1] doi: 10.1101/2025.07.31.668042 (PMC12324431; doi:10.1101/2025.07.31.668042)
Supplement: Supplement 1 [file media-1.gz › supplemental_material/src/rscape_v2.6.0.tar.gz!/rscape_v2.6.0/lib/infernal/Manuscripts/filter_thresholds/figs/tRNA_fst.pdf]

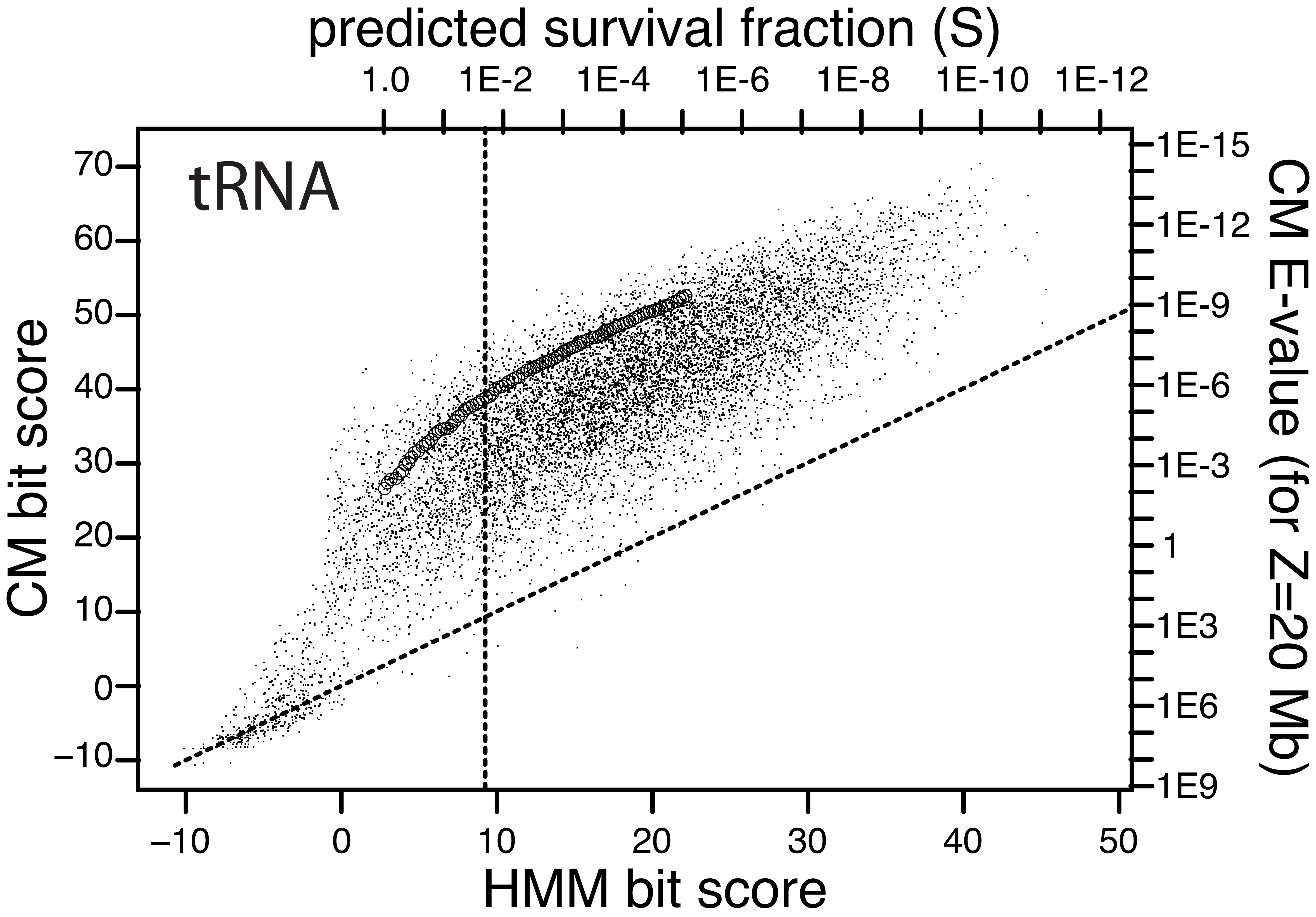
predicted survival fraction (S)
1.0
1E-2
1E-4
1E-6
1E-8
1E-10
1E-12
tRNA
CM bit score
70
60
50
40
30
20
10
0
−10
CM E-value (for Z=20 Mb)
1E-15
1E-12
1E-9
1E-6
1E-3
1
1E3
1E6
1E9
−10
0
10
20
30
40
50
HMM bit score
